# An unfavorable body composition is common in early arthritis patients: A case control study

**DOI:** 10.1371/journal.pone.0193377

**Published:** 2018-03-22

**Authors:** Samina A. Turk, Dirkjan van Schaardenburg, Maarten Boers, Sylvia de Boer, Cindy Fokker, Willem F. Lems, Michael T. Nurmohamed

**Affiliations:** 1 Amsterdam Rheumatology and Immunology Center| Reade, Amsterdam, Netherlands; 2 Amsterdam Rheumatology and Immunology Center| Academic Medical Center, Amsterdam, Netherlands; 3 Amsterdam Rheumatology and Immunology Center| VU Medical Center, Amsterdam, Netherlands; 4 Department of Epidemiology & Biostatistics, VU Medical Center, Amsterdam, Netherlands; University of Pennsylvania, UNITED STATES

## Abstract

**Background:**

An unfavorable body composition is often present in chronic arthritis patients. This unfavorable composition is a loss of muscle mass, with a stable or increased (abdominal) fat mass. Since it is unknown when this unfavorable composition develops, we compared body composition in disease-modifying antirheumatic drugs (DMARD)-naive early arthritis patients with non-arthritis controls and explored the association, in early arthritis patients, with disease activity and traditional cardiovascular risk factors.

**Methods:**

317 consecutive early arthritis patients (84% rheumatoid arthritis according to 2010 ACR/EULAR criteria) and 1268 age-/gender-/ethnicity-matched non-arthritis controls underwent a Dual-energy X-ray absorptiometry scan to assess fat percentage, fat mass index, fat mass distribution and appendicular lean (muscle) mass index. Additionally, disease activity, health assessment questionnaire (HAQ), acute phase proteins, lipid profile and blood pressure were evaluated.

**Results:**

Loss of muscle mass (corrected for age suspected muscle mass) was 4–5 times more common in early arthritis patients, with a significantly lower mean appendicular lean mass index (females 6% and males 7% lower, p<0.01). Patients had more fat distributed to the trunk (females p<0.01, males p = 0.07) and females had a 4% higher mean fat mass index (p<0.01). An unfavorable body composition was associated with a higher blood pressure and an atherogenic lipid profile. There was no relationship with disease activity, HAQ or acute phase proteins.

**Conclusion:**

Loss of muscle mass is 4–5 times more common in early arthritis patients, and is in early arthritis patients associated with a higher blood pressure and an atherogenic lipid profile. Therefore, cardiovascular risk is already increased at the clinical onset of arthritis making cardiovascular risk management necessary in early arthritis patients.

## Introduction

Inflammatory arthritis, especially rheumatoid arthritis (RA), is associated with an increased mortality [1, 2] and mainly due to cardiovascular (CV) disease[3–6]. The increased CV risk is attributed to both the presence of inflammation and an increased prevalence of traditional CV risk factors[7]. Atherosclerosis, which may already accelerate in the preclinical phase of inflammatory arthritis[5], is independently associated with a high body mass index (BMI)[8, 9], more specifically, with an excess of body fat and especially fat located on the abdomen[10]. Therefore, body composition is a better predictor for CV disease than BMI[9, 11, 12]. Body composition refers to different compartments of the body, notably fat mass and fat-free mass. Fat-free mass is also known as lean mass and includes body water, bone, organs, but primarily muscle[13]. Several studies have documented that inflammatory arthritis patients have an unfavorable body composition compared to healthy controls[14–19]. Their condition comprises a loss of skeletal muscle mass (more than suspected for their age), in the presence of stable or even increased fat mass (especially on the abdomen), resulting in a stable weight[20].

This unfavorable body composition is associated with CV comorbidity and a reduced life expectancy[21], but treat to target therapy did not improve patients’ body composition, while it did improve disease activity[14, 22, 23]. Therefore, early detection is important for preventive measures. Currently it is unknown at what point in the course of the disease an unfavorable body composition develops. Several studies found an unfavorable body composition several months after diagnosis, but no research has been performed at the onset of arthritis[14, 22]. Therefore, the objective of this study was to compare body composition between patients at the clinical onset of arthritis with the general population. Exploratory analyses were performed to determine the relation between body composition with other traditional CV risk factors and disease factors in early arthritis patients.

## Materials and methods

### Study population and assessments

The study population comprised a cohort of consecutive patients with early arthritis from the ‘Early Arthritis Cohort’ at Reade in Amsterdam, The Netherlands. This ongoing cohort includes patients of 18 years or older, with at least two swollen joints or one swollen joint with a positive rheumatoid factor (RF) and/or anti-citrullinated protein antibody (ACPA), a symptom duration of less than 2 years and no prior treatment with disease-modifying antirheumatic drugs (DMARDs). Patients with a diagnosis of crystal arthropathy, spondyloarthritis, osteoarthritis, systemic lupus erythematosus, Sjögren’s syndrome or infectious arthritis were excluded. No exclusion criteria for cardiovascular diseases were applied. Data were used of patients included from June 2008 until January 2016. Approval was obtained from Ethics Committee of the Slotervaart Hospital and Reade, Amsterdam, The Netherlands (project number P0120), and of all participating patients a written informed consent according to the Declaration of Helsinki was obtained.

Body composition was measured with the Lunar Dual-energy X-ray absorptiometry (DXA) (GE Corporate, Madison, WI, USA) before or within one month after starting treatment. Total body mass, total body fat mass, truncal and fat mass of the arms and legs were measured, whereas lean mass was used as a surrogate measure of muscle mass and is reported for the arms and legs (appendicular lean mass)[24]. Patients were interviewed to record details about symptom history, clinical characteristics, medication use and demographics, and underwent a physical examination. Disease activity was measured with the tender and swollen joints count of 28 joints and the Disease Activity Score of 28 joints with ESR (DAS28-ESR) was calculated, and physical functioning was measured by the health assessment questionnaire Disability Index(HAQ-DI)[24, 25]. Blood pressure was assessed once and measured according to the standard hospital procedures. Laboratory assessments consisted of erythrocyte sedimentation rate (ESR), RF, ACPA, and lipid profile (total cholesterol (TChol), triglycerides, low-density lipoprotein (LDL) and high-density lipoprotein (HDL)- levels).

### Control group and assessments

Early arthritis patients were matched with non-arthritis controls, from the Rotterdam Study II[26] for ethnicity (Caucasian, African or Asian), gender and age (with a range of +/-3 years) in a 1:4 ratio. The Rotterdam II open cohort study enrolls people aged 50 years or over and living in the district Ommoord of the city Rotterdam in The Netherlands and who were willing to participate. No exclusion criteria were applied. The study has been approved by the medical Ethical Committee of the Erasmus MC, Rotterdam and all participants provided written informed consent. Enrollment to the Rotterdam Study-II started in 2000. 3011 participants of the 4472 invitees were added to this cohort of which 2739 underwent a DXA scan, therefore representing a good overview of the total Rotterdam population. In Rotterdam the Lunar Prodigy device (GE Corporate, Madison, WI, USA) was used to assess body composition. Differences between the iDXA and the Prodigy device were negligible, hence cross-calibration was not necessary[27, 28].

### Statistical analyses

Data were analyzed with SPSS Version 21.0 (SPSS, Chicago, Illinois, USA). The body composition parameters which were used are BMI, fat mass index (FMI, Total body fat mass [kg]/ length^2^ [m]), percentage of fat distributed to the trunk ((Truncal fat mass [kg]/ total body fat mass [kg]) x 100%), android to gynoid fat mass ratio (Android fat mass [kg]/ gynoid fat mass [kg]) and appendicular lean mass index (ALMI, Lean mass of arms and legs [kg]/ length^2^ [m]). For the definition of obesity, the cut offs of Gallaher et al. were applied (see Table 1)[29]. From the literature no cut off values for a more than average loss of muscle mass suspected for age were available for our study population. Baumgartner et al. defined sarcopenia (low muscle mass for age) as appendicular skeletal muscle mass [kg/ height^2^ [m^2^]] more than two standard deviations (SD) below the mean of a young reference group[30]. However, our patients and control group had a mean age of 61 years, therefore we defined our own cut offs with the values of the control group. Cut off was determined on the mean minus two times the SD (see Table 2), separated for gender and divided in three ages categories: 50–59 years, 60–69 years and 70 year and older, as progressive loss of muscle mass occurs with advancing age[30]. Linear and logistic regression analysis were performed to measure the difference in body composition in early arthritis patients and the general population. To correct for multiple testing, the Benjamini-Hochberg procedure with a false discovery rate of 5% was applied[31].

**Table 1:** Definition of obesity, based on cut offs of body fat percentages by Gallaher et al[29].

	Males < 60 years	Males 60–79 years
Caucasian	>29%	>31%
African	>27%	>29%
Asian	>29%	>29%
	Females < 60 years	Females 60–79 years
Caucasian	>41%	>43%
African	>39%	>41%
Asian	>41%	>41%

**Table 2:** Cut off points of ALMI (kg/m^2^) in non-arthritis controls, for defining a low muscle mass for age, stratified for gender and age categories (mean minus two times SD).

	Number	Mean males in kg/m^2^	Cut off males in kg/m^2^
50–59 years	198	8.8 (1.0)	<6.7
60–69 years	137	8.5 (0.9)	<6.8
70–85 years	53	8.1 (0.8)	<6.6
		Mean females	Cut off females
50–59 years	453	7.0 (0.9)	<5.3
60–69 years	360	6.9 (0.8)	<5.3
70–86 years	67	7.0 (0.8)	<5.4

ALMI: appendicular lean mass index, n: number, SD: standard deviation

Next, exploratory analysis for the association of body composition and traditional risk factors in early arthritis patients were performed. Patients who used antihypertensives or statins were excluded for analyzes that involved blood pressure and cholesterol, respectively. For descriptive purposes mean (SD), median (25-75^th^ percentile) or percentages were used, where appropriate. Comparisons between groups were made with unpaired t-tests or nonparametric tests as appropriate. Linear and logistic regression exploratory analysis were performed to measure the association between body composition, disease activity and traditional cardiovascular risk factors. Correction for multiple testing was performed with the Benjamini-Hochberg procedure with a false discovery rate of 5%.

All results are presented separately for males and females, since gender was an effect modifier. Results are corrected for confounders (demographics) where appropriate.

## Results

### Patient characteristics

A total of 317 early arthritis patients (mean age 61, 69% female) were matched with 1268 non-arthritis controls, See Table 3. Almost all patients were Caucasian; five were Asian, four African. Most patients (84%) fulfilled the American College of Rheumatology (ACR)/ European League Against Rheumatism (EULAR) 2010 criteria[32]. Mean DAS28 was 5.0 (SD 1.4) points and 67% were seropositive for RF and/ or ACPA, see Table 4.

**Table 3:** Demographics.

	Early arthritis patients, n = 317	Non-arthritis controls, n = 1268
Gender, males	31	31
Age, years	61 (7)	61 (8)
Length (cm)	168 (9)	169 (9)
Weight (kg)	79 (16)	78 (15)

Results are expressed as mean (SD) or percentages. Cm: centimeters, kg: kilograms

**Table 4:** Disease activity and traditional cardiovascular risk factors in early arthritis patients, n = 317.

*Disease activity*	
DAS28	5.0 (1.4)
SJC28	6 (3–10)
TJC28	5 (2–10)
ESR in mm/hour	27 (15–46)
HAQ-DI	1 (1–2)
Symptom duration, months	7 (3–22)
RF positive	55
ACPA positive NSAID use	58 36
*Traditional cardiovascular risk factors*	
TChol, mmol/l	5.2 (1.0)
Triglycerides, mmol/l	1.2 (0.9–1.6)
HDL, mmol/l	1.4 (0.5)
LDL, mmol/l	3.2 (0.9)
TChol: HDL ratio	4.0 (1.3)
Systolic BP, mmHg	144 (22)
Diastolic BP, mmHg	84 (12)
Current smoking	25
Statin use	15
Antihypertensive use	27

Results are expressed as mean (SD), percentages, or median (25-75^th^ percentile). ACPA: anti-citrullinated protein antibody, BP: blood pressure, DAS28: disease activity score of 28 joints, ESR: erythrocyte sedimentation rate, HAQ-DI: health assessment questionnaire disability index, HDL: high-density lipoprotein, IQR: interquartile range, LDL: low-density lipoprotein, mm/hour: millimetre/hour, mmHg: millimetre mercury, mmol/l: millimole/liter, n:number, NSAID: non-steroidal anti-inflammatory drugs, RA: rheumatoid arthritis, RF: rheumatoid factor, SD: standard deviation, SJC28: swollen joint count of 28 joints, TChol: total cholesterol, TJC28: tender joint count of 28 joints

### Body composition (Fig 1, Table 5)

**Fig 1:**
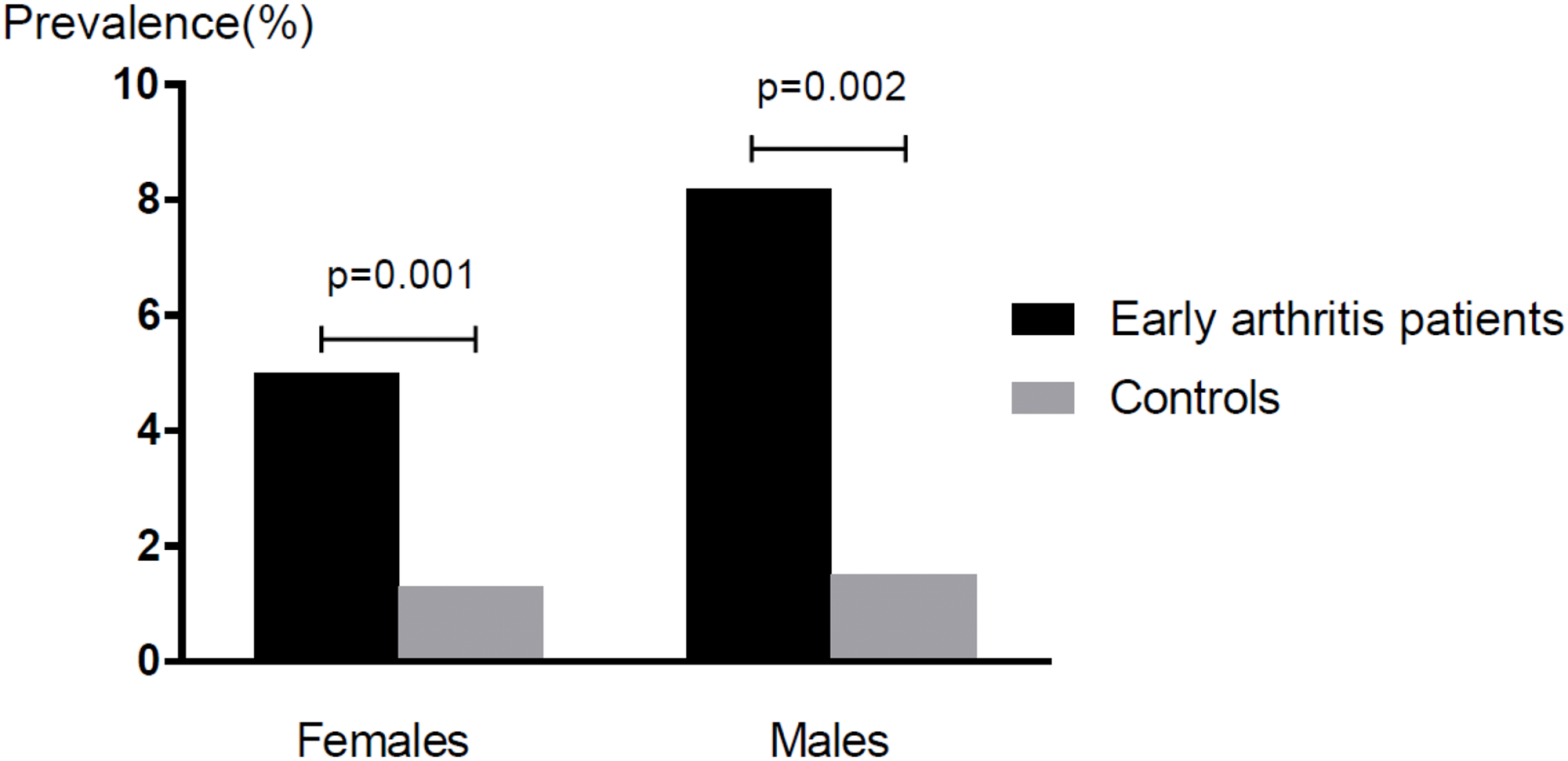
Prevalence of a low muscle mass for age in early arthritis patients compared with non-arthritis controls.

**Table 5:** Body composition indices of non-arthritis controls (females n = 880, males n = 388), and the differences of these indices with early arthritis patients (females n = 220, males n = 97), stratified for gender.

	Mean values for control females	Differences for female arthritis patients, B or OR *(CI) and p-value*	Mean values for control males	Differences for male arthritis patients, B or OR *(CI) and p-value*
BMI	27.1 (4.7)	1.0 *(0*.*26–1*.*70) 0*.008*	27.4 (3.7)	-0.3 *(-1*.*11–0*.*61) 0*.*573*
ALMI	7.0 (0.8)	-0.3 *(-0*.*44- -0*.*19) <0*.001*	8.6 (1.0)	-0.6 *(-0*.*83- -0*.*39) 0*.001*
FMI	26.6 (4.6)	1.1 *(0*.*38–1*.*78) 0*.003*	27.4 (3.6)	-0.6 *(-1*.*45–0*.*23) 0*.*154*
Android to gynoid fat mass ratio	0.5 (0.2)	<0.1 *(-0*.*04–0*.*00) 0*.*102*	0.8 (0.2)	-0.1 *(-0*.*09- -0*.*01) 0*.*029*
Body fat%	39.8 (6.4)	0.9 *(-0*.*05–1*.*92) 0*.*062*	30.7 (5.5)	-0.8 *(-2*.*07–0*.*47) 0*.*216*
% of fat distributed to the trunk	49.4 (6.5)	2.8 *(1*.*83–3*.*75) <0*.001*	57.6 (5.6)	1.2 *(-0*.*11–2*.*47) 0*.*074*
Obese	38.4	1.3 *(0*.*98–1*.*78) 0*.*068*	58	0.8 *(0*.*51–1*.*25) 0*.*324*
Low muscle mass for age	1.3	4.2 *(1*.*78–9*.*72) 0*.001*	1.5	5.7 *(1*.*94–16*.*91) 0*.002*

Results are expressed as mean (SD) or percentages and as beta (B) or odds ratio (OR) with a 95%-confidence interval (CI) and a p-value. ALMI: appendicular lean mass index, BMI: body mass index, FMI: fat mass index, n = number, RA: rheumatoid arthritis, SD: standard deviation, %: percentages

*significant results at the 0.05 false discovery rate for 18 tests, between arthritis patients and non-arthritis controls.

Compared to controls, BMI and FMI were 4% higher (p<0.01) in arthritis females, with a trend for more obesity (p = 0.07). The percentage of fat distributed to the trunk was also higher, but the android and gynoid fat mass ratio was similar. ALMI was 5–7% lower in both sexes, and a low muscle mass for their age was 4–5 times more common (in females 5.0% vs 1.3%, and in males 8.2 vs 1.5%, p<0.01). (Table 5 and Fig 1).

### Lipid levels and blood pressure and the association with body composition in early arthritis patients

Forty-six patients were on statins and 87 on antihypertensives and were excluded from these analyzes. An unfavorable body composition (higher FMI, more fat distributed to the trunk and more android fat) was associated with an unfavorable lipid profile (see S1 File). No association between ALMI and lipid levels was found.

A higher FMI and more fat distributed to the trunk were associated with a higher systolic and diastolic blood pressure. In females a higher ALMI was also related with higher blood pressures.

### Disease activity and body composition in early arthritis patients

In female patients, a higher ESR was associated with a higher FMI and patients with a longer symptom duration had more fat distributed to the trunk. In males, no association between body composition and disease activity was found. However, a longer symptom duration was related to a higher FMI and ALMI. See S2 File.

### Sensitivity analysis

Analyzes were repeated in patients who did and did not fulfill the ACR/ EULAR 2010 criteria and showed similar results (data not shown). Finally, we found that an unfavorable body composition was associated with an unfavorable lipid profile and higher blood pressure in patients without cardiovascular treatment. Therefore, analyzes including patients with statins or antihypertensives were performed, where the same results were obtained (data not shown).

## Discussion

Our study is the first to reveal that an unfavorable body composition is already present at the onset of inflammatory arthritis. A low muscle mass for age was rare, but substantially more prevalent in patients than in matched controls. In our patients, these findings could also be linked to cardiovascular risk factors, regardless of disease activity.

The mechanisms causing an unfavorable body composition in inflammatory arthritis are incompletely understood. By itself, a loss in muscle mass can result from reductions in physical activity and hormone levels as well as change in diet, all part of normal aging[20]. This also results in obesity, whereas the mean weight in the general population is nowadays already high and still increasing[33]. Whereas obesity itself is also an risk factor for the development of arthritis[34]. In inflammatory arthritis, pain, fatigue and joint stiffness further reduce physical activity[35]. Cells involved in inflammation produce proinflammatory molecules, like IL-6, that increase muscle metabolism with subsequent muscle wasting[36]. Furthermore, arthritis is associated with an insulin resistance, which may lead to muscle protein degradation[37].

Our findings are in line with the results observed in established arthritis[16, 19]. In established arthritis patients an association between body composition and glucocorticoid use was found[19, 38, 39]. Only a minority of the arthritis patients received steroids during a few days. As our population underwent the DXA scan before or within one month after the start of treatment, glucocorticoid use was of minimum influence[40]. Hence, altogether the influence of steroids appears to be negligible.

In the present study, the number of early arthritis patients with a low muscle mass for age was low, which might explain that no associations between disease activity and an unfavorable body composition could be demonstrated. The relation between different aspects of disease activity and components of body composition were inconclusive. Nevertheless, a longer symptom duration was associated with an increased FMI and ALMI, however due to the small beta coefficients this was not clinical relevant (every one month increase in symptom duration, was associated with an increase in FMI and ALMI of 0.04 and 0.01, respectively). Moreover, in females a higher ESR was associated with an increase in FMI (10 points increase in ESR was associated with a 0.4 points increase in FMI). However, ESR might not be representative of intramuscular activity, therefore we recommend for future research to further analyse the association between disease activity and an unfavorable body composition by extending the measurements with CRP and IL-6 levels[19].

In early arthritis patients an unfavorable body composition was associated with a higher blood pressure and higher lipid levels (with lower HDL levels), which is according to previous literature, similar to the general population[41–43]. However, as inflammation generally leads to a decreased TC and HDL level, but an increased TC:HDL ratio, it is in RA patients difficult to interpret the lipid levels[44, 45]. The combination of an unfavorable body composition, hypertension and an atherogenic lipid profile might be a clustering of risk factors, known as metabolic syndrome, as overweight is often associated with hypertension and hypercholesterolemia[46]. This association between an unfavorable body composition and traditional risk factors might help explain the increased prevalence of CV disease in arthritis patients. Van Halm et al. already showed a more atherogenic lipid profile in blood donors who later developed RA, which was partly explained by the presence of inflammation[47, 48]. There are a number of factors that are associated with both body composition, lipid profile and blood pressure, including lifestyle factors such as a diet high in fat, sugar and sodium, insufficient physical activity and family history[49]. Hormones derived from adipose tissue have also been linked to an increased blood pressure, include leptin and adiponectin[50]. As inflammatory arthritis and CV diseases are multifactorial disorders, overlapping risk factors and a shared etiology for the development of both diseases have to be considered. Smoking and metabolic syndrome are important risk factors for the development of both arthritis and CV disease[51, 52]. The development of arthritis and CV disease are also, partially, explained to common susceptibility genes, however must more research on this area is necessary[52]. A suggested shared etiology is periodontal disease which is generated by microorganisms, like Porphyromonas gingivalis (Pg). In RA patients an antibody response to Pg is common and Pg also contributes to the pathogenesis of atherosclerosis[49, 53, 54]. Future studies are needed to determine if an unfavorable body composition already exists before the onset of arthritis. Hence, DXA scans should be performed in patients with a high risk for developing RA. This will further define the optimal moment for a DXA scan and might give clues how we can prevent this unfavorable body composition, as RA treatment itself does not appear to improve body composition[14, 22, 23].

The strengths of this study include a good match with an excellent population cohort, the large sample size for both males and females. Unfortunately, no widely accepted definition of a low muscle mass for age exists[55]. Nevertheless, both muscle mass and muscle strength are probably a main component. A limitation of the current study is that no assessments of muscle strength and physical performance were done. Therefore, we defined our own definition for sarcopenia low muscle mass for age, based on the muscle mass of the non-arthritis control group, so our main question could still be answered as we focussed on the difference between patients and non-arthritis controls. Another limitation is the possibility of selection bias, as the selection was inherently different between arthritis patients and non-arthritis controls. The subjects were selected from different locations, which can give differences in demographics. However, we matched on age, gender and ethnicity and added the values of body composition after the matching to limit this bias. Another aspect are the DXA machines that were utilized. The arthritis patients and the non-arthritis controls are measured in different DXA machines and the machines were not directly calibrated.

## Conclusion

In conclusion, unfavorable body composition occurs early in the development of inflammatory arthritis, and this was associated with an increased CV risk. Therefore, we suggest that CV risk management should already be initiated at disease onset.

## Supporting information

S1 File Linear regression analyses between traditional cardiovascular risk factors and body composition in early arthritis patients, stratified for gender and corrected for age, smoking status and NSAID use. (PDF)

S2 File Linear regression analyses between disease activity and body composition in early arthritis patients, stratified for gender and corrected for age and smoking status. (PDF)
