# Supplementary material for: An unfavorable body composition is common in early arthritis patients: A case control study
Source: PLoS One. 2018 Mar 22;13(3):e0193377. doi: 10.1371/journal.pone.0193377 (PMC5863963; doi:10.1371/journal.pone.0193377)
Supplement: S1 File — (PDF) [file pone.0193377.s001.pdf]

S1 File. Linear regression analyses between traditional cardiovascular risk factors and body composition in early arthritis patients, stratified for gender and corrected for age, smoking status and NSAID use.

Females, n=220

	FMI	Percentages of fat distributed to the trunk	Android to gynoid fat mass ratio	ALMI
	B<br>*(CI) and p-value*	B<br>*(CI) and p-value*	B<br>*(CI) and p-value*	B<br>*(CI) and p-value*
Systolic BP, mmHg‡	0.08<br>*(0.04-0.13) <0.001**	0.08<br>*(0.03-0.13) 0.004**	<0.01<br>*(0.00-0.00) 0.003**	0.01<br>*(0.00-0.02) 0.004**
Diastolic BP, mmHg‡	0.16<br>*(0.10-0.23) <0.001**	0.12<br>*(0.04-0.21) 0.004**	<0.01<br>*(0.00-0.01) 0.003**	0.02<br>*(0.01-0.03) 0.008**
Total Cholesterol, mmol/l†	-0.12<br>*(-0.90-0.66) 0.759*	1.18<br>*(0.27-2.09) 0.012**	0.03<br>*(0.01-0.06) 0.003**	-0.09<br>*(-0.23-0.04) 0.168*
Triglycerides, mmol/l†	0.98<br>*(-0.47-2.44) 0.182*	4.06<br>*(2.42-5.70) <0.001**	0.12<br>*(0.08-0.16)<0.001**	-0.02<br>*(-0.26-0.22) 0.847*
HDL, mmol/l†	-2.37<br>*(-4.2-0.61) 0.009**	-3.18<br>*(-5.27- -1.01) 0.003**	-0.09<br>*(-0.14- -0.04)<0.001**	-0.31<br>*(-0.62-0.00) 0.050*
LDL, mmol/l†	0.31<br>*(-0.54-1.17) 0.472*	1.46<br>*(0.47-2.45) 0.004**	0.04<br>*(0.02-0.06), 0.001**	-0.03<br>*(-0.18-0.12) 0.732*
TChol: HDL ratio†	0.38<br>*(-0.25-1.01) 0.234*	1.47<br>*(0.76-2.19) <0.001**	0.04<br>*(0.02-0.06)<0.001**	0.03<br>*(-0.08-0.13) 0.649*

Males, n=97

	FMI	Percentages of fat distributed to the trunk	Android to gynoid fat mass ratio	ALMI
	B<br>*(CI) and p-value*	B<br>*(CI) and p-value*	B<br>*(CI) and p-value*	B<br>*(CI) and p-value*
Systolic BP, mmHg ‡	0.06<br>*(-0.01-0.12) 0.071*	0.06<br>*(-0.03-0.15) 0.182*	<0.01<br>*(0.00-0.01) 0.137*	0.01<br>*(-0.01-0.03) 0.168*
Diastolic BP, mmHg‡	0.14<br>*(0.01-0.27) 0.040*	0.10<br>*(-0.09-0.29) 0.276*	<0.01<br>*(0.00-0.01) 0.240*	0.02<br>*(-0.01-0.05) 0.236*
Total Cholesterol, mmol/l †	0.29<br>*(-0.90-1.48) 0.630*	2.01<br>*(0.30-3.73) 0.022**	0.07<br>*(0.01-0.12) 0.013**	-0.01<br>*(-0.33-0.30) 0.929*
Triglycerides, mmol/l†	2.09<br>*(0.27-3.91) 0.025**	5.81<br>*(3.29-8.34) <0.001**	0.15<br>*(0.07-0.22) <0.001**	-0.10<br>*(-0.60-0.39) 0.677*
HDL, mmol/l†	-3.11<br>*(-6.22-0.01) 0.050*	-4.53<br>*(-9.17-0.12) 0.056*	-0.15<br>*(-0.28- -0.01) 0.040*	-0.23<br>*(-1.07-0.60) 0.577*
LDL, mmol/l†	0.55<br>*(-0.86-1.95) 0.437*	2.23<br>*(0.24-4.21) 0.028*	0.08<br>*(0.02-0.14) 0.007**	0.07<br>*(-0.30-0.43) 0.723*
TChol: HDL ratio†	1.04<br>*(0.25-1.82) 0.011**	1.96<br>*(0.82-3.10) 0.001**	0.06<br>*(0.03-0.10) <0.001**	0.10<br>*(-0.11-0.32) 0.351*

ALMI: appendicular lean mass index, B: beta (1 point increase in blood pressure or lipid profile is X change in FMI, percentages of fat distributed to the trunk, android to gynoid fat mass ratio or LMI), BP: blood pressure, CI: confidence interval, FMI: fat mass index, HDL: high-density lipoprotein, LDL: low-density lipoprotein, mmHg: millimetre mercury, mmol/l: millimole/liter, NSAID: non-steroidal anti-inflammatory drugs, TChol: total cholesterol
‡Patients without antihypertensives
†Patients without statins
*significant results at the 0.05 false discovery rate for 56 tests.
