# Supplementary material for: An unfavorable body composition is common in early arthritis patients: A case control study
Source: PLoS One. 2018 Mar 22;13(3):e0193377. doi: 10.1371/journal.pone.0193377 (PMC5863963; doi:10.1371/journal.pone.0193377)
Supplement: S2 File — (PDF) [file pone.0193377.s002.pdf]

S2 File. Linear regression analyses between disease activity and body composition in early arthritis patients, stratified for gender and corrected for age and smoking status.

	FMI	Percentages of fat distributed to the trunk	Android to gynoid fat mass ratio	ALMI
Females, n=220	B<br>*(CI) and p-value*	B<br>*(CI) and p-value*	B<br>*(CI) and p-value*	B<br>*(CI) and p-value*
ESR in mm/hour (median, IQR)	0.04<br>*(0.00-0.07) 0.028*	0.01<br>*(-0.03-0.04) 0.797*	<0.01<br>*(0.00-0.00) 0.861*	<0.01<br>*(0.00-0.01) 0.387*
DAS28 (mean, SD)	0.33<br>*(-0.17-0.83) 0.194*	-0.39<br>*(-0.98-0.20) 0.190*	-0.01<br>*(-0.02-0.01) 0.343*	-0.01<br>*(-0.10-0.081) 0.869*
SJC38 (median, IQR)	-0.09<br>*(-0.20-0.01) 0.089*	-0.13<br>*(-0.26- -0.01) 0.040*	<0.01<br>*(-0.01-0.00) 0.030*	-0.01<br>*(-0.03-0.01) 0.195*
TJC38 (median, IQR)	0.06<br>*(-0.03-0.15) 0.204*	-0.03<br>*(-0.14-0.07) 0.534*	<0.01<br>*(0.00-0.00) 0.894*	<0.01<br>*(-0.01-0.05) 0.403*
RF positive (n,%)	0.44<br>*(0.99-1.87) 0.546*	0.43<br>*(-1.27-2.13) 0.616*	0.02<br>*(-0.02-0.06) 0.400*	0.03<br>*(-0.22-0.28) 0.812*
ACPA positive (n,%)	-0.04<br>*(-1.49-1.42) 0.963*	0.89<br>*(-0.83-2.61) 0.310*	0.02<br>*(-0.02-0.06) 0.389*	-0.07<br>*(-0.33-0.19) 0.238*
HAQ-DI (median, IQR)	0.99<br>*(-0.01-1.98) 0.052*	-0.92<br>*(-2.08-0.24) 0.120*	-0.01<br>*(-0.04-0.02) 0.387*	0.04<br>*(-0.14-0.22) 0.654*
Symptom duration, months (median, IQR)	0.01<br>*(0.00-0.02) 0.068*	0.01<br>*(0.00-0.03) 0.020*	<0.01<br>*(0.00-0.00) 0.478*	<0.01<br>*(0.00-0.00) 0.068*
Males, n=97				
ESR in mm/hour (median, IQR)	<0.01<br>*(-0.04-0.04) 0.969*	-0.04<br>*(-0.09-0.02) 0.173*	<0.01<br>*(0.00-0.00) 0.615*	<0.01<br>*(-0.01-0.01) 0.543*
DAS28 (mean, SD)	0.48<br>*(-0.15-1.10) 0.132*	-0.10<br>*(-1.02-0.83) 0.834*	<0.01<br>*(-0.03-0.03) 0.935*	0.03<br>*(-0.13-0.19) 0.694*
SJC38 (median, IQR)	0.05<br>*(-0.06-0.17) 0.365*	<0.01<br>*(-0.17-0.17) 0.998*	<0.01<br>*(-0.01-0.00) 0.486*	0.02<br>*(-0.01-0.05) 0.189*
TJC38 (median, IQR)	0.10<br>*(0.00-0.21) 0.204*	-0.02<br>*(-0.17-0.14) 0.844*	<0.01<br>*(-0.01-0.00) 0.655*	0.02<br>*(-0.01-0.05) 0.134*
RF positive (n,%)	1.04<br>*(-0.79-2.88) 0.261*	-2.24<br>*(-4.90-0.43) 0.098*	-0.04<br>*(-0.12-0.04) 0.352*	0.36<br>*(-0.11-0.82) 0.132*
ACPA positive (n,%)	-0.20<br>*(-2.06-1.65) 0.829*	-2.04<br>*(-4.72-0.64) 0.135*	-0.07<br>*(-0.15-0.02) 0.109*	-0.16<br>*(-0.63-0.32) 0.511*
HAQ-DI (median, IQR)	0.52<br>*(-0.76-1.79) 0.422*	0.18<br>*(-1.62-1.98) 0.841*	0.02<br>*(-0.03-0.08) 0.443*	0.05<br>*(-0.27-0.37) 0.761*
Symptom duration, months (median, IQR)	0.04<br>*(0.00-0.07) 0.044*	0.01<br>*(-0.04-0.06) 0.744*	<0.01<br>*(0.00-0.00) 0.478*	0.01<br>*(0.00-0.02) 0.024*

ACPA: anti-citrullinated protein antibody, ALMI: appendicular lean mass index, B: beta (1 point increase in parameters of disease activity is X change in FMI, percentages of fat distributed to the trunk, android to gynoid fat mass ratio or LMI), CI: confidence interval, DAS28: disease activity score of 28 joints, ESR: erythrocyte sedimentation rate, FMI: fat mass index, HAQ-DI: health assessment questionnaire disability index, mm/hour: millimetre/hour, n:number, RF: rheumatoid factor, SJC38: swollen joint count of 38 joints, TJC38: tender joint count of 38 joints
*significant results at the 0.05 false discovery rate for 64 tests.
